# Injectable MXene/Ag-HA composite hydrogel for enhanced alveolar bone healing and mechanistic study

**DOI:** 10.3389/fbioe.2024.1485437

**Published:** 2024-12-11

**Authors:** Jialing Li, Zilu Fan, Zhenju Guan, Jianping Ruan

**Affiliations:** ^1^ Key Laboratory of Shaanxi Province for Craniofacial Precision Medicine Research, College of Stomatology, Xi’an Jiaotong University, Xi’an, Shaanxi, China; ^2^ Nanchong Central Hospital (Nanchong Hospital of Beijing Anzhen Hospital,Capital Medical University), the Second Clinical College of North Sichuan Medical College, Nanchong, Sichuan, China; ^3^ Nanchong Mental Health Center Of Sichuan Province, Nanchong Second People’s Hospital, Nanchong Senior Hospital, Nanchong, Sichuan, China; ^4^ Center of Oral Public Health, College of Stomatology, Xi’an Jiaotong University, Xi’an, Shaanxi, China

**Keywords:** alveolar bone defect, osteoinductivity, MXene, hydrogel, osteogenesis

## Abstract

**Introduction:**

Alveolar bone defects pose significant challenges in dentistry. Due to the complexity of alveolar bone anatomy and insufficient repair mechanisms, large bone defects are difficult for the body to heal naturally. Clinical treatment typically involves the use of bone substitute materials. However, current substitutes often suffer from limitations such as insufficient osteoinductivity, rapid degradation, inflammatory responses, and poor mechanical properties. Additionally, the irregular morphology of alveolar bone defects complicates the application of solid bone substitutes, potentially leading to secondary damage at the repair site.

**Methods:**

To address these challenges, this study introduces an innovative approach by integrating MXene nanomaterials into Ag-HA/GelMA hydrogels to create an injectable MXene/Ag-HA composite hydrogel. MXene nanomaterials are renowned for their excellent biocompatibility, antibacterial properties, and mechanical strength.

**Results:**

The results indicate that the MXene/Ag-HA composite hydrogel exhibits satisfactory mechanical and biological properties. Specifically, it demonstrates excellent antibacterial, antioxidant, and osteogenic activities. Gene expression analysis further reveals that the MXene composite hydrogel promotes osteogenesis by regulating the expression of Dmp1 and Dusp1.

**Discussion:**

The findings of this study suggest that the MXene/Ag-HA composite hydrogel is a promising candidate for alveolar bone repair and regeneration. The integration of MXene nanomaterials into the hydrogel enhances its mechanical and biological properties, making it well-suited for the treatment of irregular alveolar bone defects. Furthermore, the study underscores the vast potential of MXene nanomaterials in the biomedical field, hinting at potential applications beyond alveolar bone repair.

## 1 Introduction

Alveolar bone defects refer to the loss or destruction of alveolar bone tissue, most commonly caused by chronic alveolar bone resorption due to periodontitis (Li et al., 2023). Due to the complex anatomy of the alveolar bone and insufficient repair mechanisms, when bone defects exceed a certain size, the body struggles to self-repair (Ramezanzade et al., 2023; Wang et al., 2022), thus clinical intervention is necessitated.

At present, the application of artificial bone replacement in treating alveolar bone defects is becoming increasingly widespread. Ideal artificial bone substitute materials must possess various properties such as favorable biodegradability as well as biocompatibility, concordant mechanical strength to bone tissue, and a porous network construction conducive for the cell growth (Iocca et al., 2017; Faour et al., 2011; Toosi et al., 2024). Hydroxyapatite (HA), a primary component of natural bone, can increase local Ca^2+^ concentration, thereby activating osteoblast proliferation and promoting the discrepancy and growth of mesenchymal stem cells (MSCs) (Zhao et al., 2017; Ren et al., 2023). Due to its non-immunogenicity, acceptable osteoconductivity, and bioactivity, HA has been frequently applied in bone repair (Yu et al., 2022; Shi et al., 2021). Interestingly, the crystal structure of HA facilitates various ion substitutions. This ion exchange affects the concentration, size, and interaction type of the HA lattice, improving its physicochemical properties by altering its electron density and surface conditions, thereby optimizing its performance (Daniel Arcos and Vallet-Regí, 2020). Within a low silver loading range of 0.5%–2% (wt), Ag-HA exhibits excellent antibacterial activity along with outstanding biocompatibility and bone-binding capacity (Mirzaee et al., 2016).

Gelatin methacryloyl (GelMA) is a typical synthetic hydrogel formulation widely employed in biomedical fields (Kurian et al., 2022) with low antigenicity, significant biocompatibility, and cellular response properties. It can be crosslinked and solidified into a gel in the presence of a photoinitiator due to the double-bond modified gelatin feature under UV or visible light illumination. While introduced as a scaffold in bone repair systems, GelMA owns synthetic and natural characteristics of biomaterials (Yue et al., 2015; Zhang et al., 2020), its three-dimensional structure also benefits for the cell growth and differentiation.

MXene, a two-dimensional transition metal carbide nitride discovered and synthesized in 2011, exhibits excellent photothermal effects, bioimaging capabilities, conductivity, biocompatibility, and antibacterial properties, making it widely popular in the design of biomaterials and extensively applied in photodynamic therapy, biosensors, and drug delivery systems (Gazzi et al., 2019; Sinha et al., 2018; Ye et al., 2024). It also demonstrates a wide range of applications in the field of osteogenesis; its excellent antibacterial performance and ability to promote bone regeneration and angiogenesis have been verified. Furthermore, due to its efficient photothermal conversion and stimulation of bone regeneration, it has also been explored for applications in osteosarcoma (Yin et al., 2021; Wang et al., 2023).

This study was dedicated to developing an innovative injectable hydrogel for alveolar bone regeneration. In constructing the composite hydrogel, we chose GelMA as the three-dimensional scaffold material, which provided a suitable porous structure that promoted cell proliferation and differentiation. We incorporated a low concentration of Ag-HA into GelMA and innovatively introduced MXene materials. These additions not only overcame the high brittleness of Ag-HA but also significantly enhanced the hydrogel’s antibacterial, mechanical, and osteogenic properties. Through comprehensive physical characterization and *in vitro* and *in vivo* anti-inflammatory osteogenesis experiments, the results demonstrated that the 0.3% MXene/Ag-HA composite hydrogel exhibited excellent mechanical properties, biodegradability, biocompatibility, anti-inflammatory, antioxidant, and osteogenic properties. Therefore, this composite hydrogel was expected to become an ideal material in the next-generation of bone repair and regeneration, offering a new solution for treating bone diseases such as alveolar bone defects.

## 2 Materials and methods

### 2.1 Cells and materials

For the Cell culture, MC3T3-E1 and RAW 264.7 cells (acquired from the Kunming Cell Bank, Chinese Academy of Sciences) were cultured in a 5% CO₂ humidified incubator under 37°C. For the materials, the following reagents were purchased from Sigma-Aldrich: (NH_4_) H_2_PO_4_, Ca(NO_3_)_2_, HCl, AgNO_3_, NH_4_OH, gelatin, methacrylic anhydride, LAP, and Ti_3_AlC_2_. The CCK-8 kit and qPCR kit were obtained from Vazyme Biotech. The live/dead cell staining kit and ROS detection kit were purchased from Beyotime Biotechnology. Antibodies for CD206, iNOS, OPN, OCN, and VEGF were acquired from Abcam. The ALP staining kit and ARS staining kit were also obtained from Sigma-Aldrich.

### 2.2 Preparation of MXene/Ag-HA composite hydrogel

10 g of gelatin were weighed and placed into a round-bottom flask, followed by the addition of 100 mL PBS solution. The mixture was stirred at 45°C in a water bath for 12 h. Then, 6 g of methacrylic anhydride were added dropwise, and the reaction continued for 2 h. Then, the supernatant was collected for dialysis after the centrifuging the mixture. Upon completion of dialysis, the material was freeze-dried to obtain a 10% concentration of GelMA hydrogel, which was stored at 4°C in the dark.

To prepare Ag-HA, using the chemical formula Ag_x_Ca_10-x_ (PO)_4_(OH)_2_, a certain concentration of (NH_4_)H_2_PO_4_ and Ca(NO_3_)_2_ solution was mixed. AgNO_3_ was dissolved in the Ca(NO_3_)_2_ solution to achieve a 1% Ag content, where Ag^+^ replaced Ca^2+^ in the reaction, preparing Ag-loaded HA. Subsequently, the prepared Ag-HA was added to the GelMA solution at 3 w/v% of concentration, along with 0.25 w/v% for LAP photoinitiator. The mixture was thoroughly stirred to ensure uniform dispersion, forming GelMA/HA composite hydrogel (Abbreviated as AG).

Next, TI_3_AlC_2_ was etched with HCl to synthesize the MXene required for the experiment. MXene was then mixed with AG hydrogel at different mass molar ratios, thoroughly stirred to achieve homogeneity, and resulted in MXene/Ag-HA/GelMA composite hydrogels with MXene proportions of 0.1%, 0.3%, and 0.5% (Abbreviated as MAG).

### 2.3 Characterization analysis

The samples were divided into five groups: Control groups included GelMA group and AG group, target groups included 0.1%, 0.3%, and 0.5% of MAG. The hydrogel solutions were then injected into cylindrical molds with a height of 15 mm (n = 3 per group) and a diameter of 10 mm, respectively. The composite hydrogels were treated under 405 nm blue light illumination for 30 s to allow for setting. After curing, the samples were frozen in liquid nitrogen for 1 min and then freeze-dried for 48 h to prepare them for subsequent experiments. The morphology, elemental composition, mechanical properties, and conductivity of the hydrogels in each group were investigated and discussed. Results are presented as mean ± standard deviation. Quantitative data were analyzed using one-way ANOVA with GraphPad Prism 9.0, and a p-value of <0.05 was considered statistically significant.

### 2.4 Evaluation of the *in vitro* biological properties of hydrogels

#### 2.4.1 Biocompatibility

The hydrogels seeded with MC3T3-E1 cells were kept in 24-well plates, and the culture medium was changed at an interval of 2 days. On the first, fourth, and seventh days, three samples from each group were taken, and 200 µL of culture medium containing 10% CCK-8 was introduced to each well. After incubating the samples for 2 h at 37°C, 100 µL of the corresponding incubation solution was then moved to a 96-well plate. Additionally, on the fourth day, live/dead cell staining was performed, and the samples were investigated under a fluorescence microscope. The optical density (OD) was measured at 450 nm.

#### 2.4.2 Antibacterial properties

MXene can rapidly absorb light energy and convert it into heat (Parajuli et al., 2022), significantly increasing its surface temperature, which is beneficial for antibacterial activity. In this study, NIR illumination was applied for 4 min, and the temperature change of the hydrogel was recorded to explore the photothermal conversion potential of the composite hydrogel after the addition of MXene. Subsequently, the antibacterial performance of various hydrogel groups against *staphylococcus aureus* was evaluated using the plate-counting method. Selected samples were divided into two groups: one group received NIR illumination, while the other group was not proceeded. The samples were then co-cultured with bacterial suspension for 12 h and incubated for 48 h under 37°C. Bacterial colony growth can be observed after incubation, moreover, the antibacterial performance of each hydrogel group was compared.

#### 2.4.3 Anti-inflammatory and antioxidant properties

The samples from each group were co-cultured with RAW 264.7 for 48 h, followed by fixation with 4% paraformaldehyde. After fixation, the cells were blocked and subjected to antibody staining, and the signals for CD206 and iNOS were detected using a fluorescence filter. Subsequently, the samples were co-cultured with MC3T3-E1 cells for 48 h to measure the ROS levels.

#### 2.4.4 Osteogenic performance *in Vitro*

To observe the angiogenesis of MC3T3-E1 co-cultured with hydrogels lasting for 6 h, immunofluorescence detection of VEGF, OCN, and OPN was conducted at specific time points (2 or 4 days) to assess the expression of osteogenic proteins. Additionally, ALP as well as ARS staining were employed to detect osteogenic differentiation and calcium nodule deposition, while qPCR measured the expression of the osteogenic-corresponded genes OPN, OCN and ALP in MC3T3-E1 cells. These experiments comprehensively evaluated the osteogenic performance of the different hydrogel groups.

#### 2.4.5 *In vivo* biological performance of hydrogels

A rat calvarial defect model with a diameter of about 5 mm on both sides of the skull was established to simulate a bone defect environment, into which hydrogels with different materials were implanted. At 4 and 8 weeks of post-surgery, the influence of these materials on bone regeneration as well as osseointegration were evaluated via Micro-CT scanning, immunohistochemical staining, and histological analysis.

#### 2.4.6 Investigation of the molecular mechanisms and signaling pathways of osteogenesis regulated by MAG composite hydrogels

RNA-seq sequencing combined with bioinformatics analysis was used to screen for differentially expressed genes in rat MC3T3-E1 cells treated with MAG and AG hydrogels. The relevant osteogenic mechanisms were explored based on these findings.

## 3 Results and discussion

### 3.1 Synthesis of MXene

**FIGURE 1:**
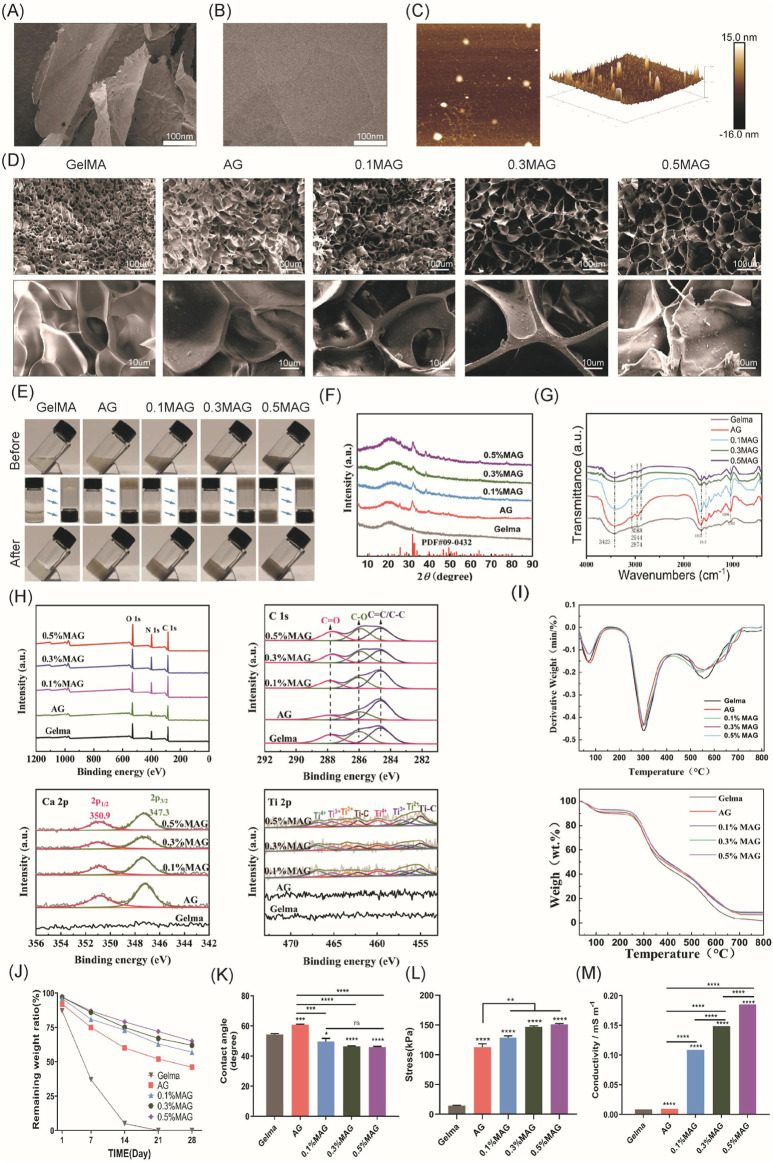
**(A)** SEM images, **(B)** TEM images, and **(C)** AFM images of MXene; **(D)** SEM images of each hydrogel group; **(E)** Macroscopic images of each hydrogel group before and after exposure to blue light; **(F)** XRD spectra of each hydrogel group; **(G)** FT-IR spectra of each hydrogel group; **(H)** XPS spectra and peak fitting of each hydrogel group; **(I)** TGA and DTG curves of each hydrogel group; **(J)** *In vitro* degradation curves of each hydrogel group; **(K)** Contact angle analysis of each hydrogel group; **(L)** Compressive strength results of each hydrogel group.; **(M)** Conductivity of each hydrogel group.

As depicted in Figure 1, SEM (Scanning electron microscope) image from Figure 1A and TEM (Transmission electron microscope) image from Figure 1B reveal a stacked flake-like structure forming the multilayered configuration. AFM image from Figure 1C observations of the synthesized MXene material’s surface morphology also indicate that the successful preparation of multilayer MXene, with a sample thickness of approximately 15 nm.

### 3.2 Microstructure of hydrogels

The microstructure of hydrogels possesses a significant impact on their overall performance. The internal structure is obliged to not only provide stable support but also create an appropriate micro-environment for cell proliferation. In this experiment, SEM was employed to observe the surface morphology for the hydrogels in each group, as exhibited in Figure 1D. With the addition of Ag-HA and MXene, the pore structure of GelMA changed significantly, with thicker and rougher pore walls, which markedly improved the mechanical properties of the hydrogels. Furthermore, the widened pore size facilitated cell proliferation and material exchange, and the connectivity of the materials was also enhanced. Both of the 0.3% and 0.5% MAG groups demonstrated larger pore sizes; However, in the 0.5% group, the increased MXene content resulted in more graphene-like wrinkled layered structures on some pore walls, blocking the pore entrances, which might adversely affect cell proliferation.

### 3.3 Macroscopic Appearance and injectability

As shown in Figure 1E, the GelMA group is visually observed as a colorless and transparent gel. With the addition of Ag-HA, the color changes to light gray, and upon further addition of MXene, the color deepens to dark gray. Injectable hydrogels, due to their tunable properties, have become one of the most attractive types of hydrogels (Lin et al., 2018; Li et al., 2021). To verify the injectability of the samples in this experiment, we recorded the state of the hydrogels before and after exposure to 405 nm blue light. Before illumination, the materials in the bottles were in a liquid state and could flow, with the liquid surface parallel to the ground when the bottle was tilted. After illumination, due to the crosslinking reaction, the materials in the bottles became solid; when the bottle was tilted or inverted, the shape of solid material was retained. This result indicated that the fluidity of the MAG hydrogel was crucial for filling complex alveolar bone defects.

### 3.4 Composition analysis of hydrogel

To investigate the chemical composition of various hydrogel groups, a series of tests including XRD (X-ray diffraction), FT-IR, XPS (X-ray photoelectron spectroscopy), and thermogravimetric analysis were conducted.As shown in Figure 1F . In the XRD measurement, GelMA combined with Ag-HA showed characteristic diffraction peaks of HA at 2*θ* = 31°–33°, indicating successful incorporation of HA. However, no distinct MXene peaks were observed in the MAG hydrogel group, likely due to the low concentration and good dispersion of MXene within the hydrogel.

As shown in Figure 1G, in the infrared spectra of the GelMA samples, a series of infrared vibration peaks were observed. The 3,423 cm^−1^ and 3,068 cm^−1^ peaks indicate the stretching vibrations of the N-H bonds in the amide A and B bands, while the 2,944 cm^−1^ and 2,874 cm^−1^ peak are attributed to C-H vibrations at different positions on the GelMA surface. The peak at 1,635 cm^−1^ mainly results from the C=O vibration within the conjugated amide groups, and the peak at 1,543 cm^−1^ corresponds to the coupled C-N stretching vibration as well as N-H bending vibration. These peaks confirm the presence of GelMA. After combining GelMA with Ag-HA, new peaks appeared at 1,035 cm^-1^, 871 cm^-1^, 603 cm^-1^, and 564 cm^-1^, corresponding to the characteristic absorption peaks of the PO_4_^3−^ group, suggesting the presence of hydroxyapatite. Upon further combination with different concentrations of MXene, the characteristic peaks of GelMA and hydroxyapatite remained, while the peak intensity at 3,423 cm^−1^ gradually decreased, and the peak at 1,085 cm^-1^ disappeared, leaving the C-O-C stretching peak at 1,035 cm^−1^. This demonstrates that GelMA is connected to the MXene surface’s oxygen-containing functional groups via amide bonds. Furthermore, no other extraneous peaks were observed, indicating the successful combination of these materials while maintaining their original chemical structures.

As shown in Figure 1H, The XPS survey spectrum revealed characteristic peaks of C, N, and O elements. However, due to the low concentration and even dispersion of Ti in the hydrogel, its presence was not well reflected in the survey spectrum. We performed peak fitting for C, Ca, and Ti elements, as shown in the figures. With the increase for MXene concentration, the intensity of the C-O peak at 287.8 eV as well as the C=O peak at 286 eV was also improved, and these characteristic peaks shifted towards lower binding energies, specifically to 287.6 eV and 285.9 eV, which indicated that in the MAG hydrogel, the rich functional groups on the MXene surface chemically reacted and interacted with the carbon and hydroxyl groups in GelMA, forming enriched C-O and C=O bonds. When fitting the Ca peak, the MAG group showed specific peaks at 347.3 eV and 350.9 eV corresponding to Ca 2p_3/2_ and Ca 2p_1/2_, respectively, while this observation was not detected in the GelMA group, confirming the successful incorporation of hydroxyapatite into the composite hydrogel. In the Ti peak fitting, the MAG group exhibited Ti-C bonds, with increasing peak intensity correlating with the MXene concentration, demonstrating the presence of MXene in the MAG composite hydrogel and its effective bonding with the other components via Ti-C bonds.

As shown in Figure 1I, Thermogravimetric analysis is used to distinguish the organic and inorganic components of the samples. The experiments showed that the weight of the five hydrogel groups decreased with increasing temperature from room temperature to 800°C. Moreover, the organic components in the hydrogels rapidly decomposed at 300°C and they were completely decomposed by approximately 600°C. Ultimately, the MAG group retained the highest weight close to 10%, while the GelMA group retained less than 2%, illustrating the successful preparation of composite hydrogels with varying mass percentages of MXene.

### 3.5 Mechanical properties of hydrogel

Appropriate degradation of bone regeneration scaffolds was crucial for their temporary support function. Herein, we evaluated the *in vitro* degradation performance for each group of composite hydrogels via immersed in PBS solution, and the degradation curves were shown in Figure 1J. With the addition of low concentrations of HA and MXene, the hydrogels remained essentially intact in the early 1–2 weeks, providing sufficient mechanical support to stabilize the wound site and support cell adhesion and early tissue repair. By the 3rd to 4th week, MAG hydrogels gradually degraded, providing space for osteogenic cells and newly formed matrix. As exhibited in Figure 1K, for the hydrogels in 5 groups, the MAG group exhibited a lower water contact angle, indicating good wettability of the material with cells or organisms. This was beneficial for cell adhesion, growth, and tissue repair.

Compression tests were used to evaluate the compressive performance of hydrogels, aiming to understand their deformation characteristics and strength under pressure. As shown in Figure 1L, the maximum compressive stress reached 151.01 ± 1.52 kPa after the addition of HA and MXene, which was more than 10 times higher compared to the GelMA group, significantly enhancing the compressive strength of the composite hydrogel. This enhancement can be attributed to the filling effect and physical crosslinking of HA and MXene, optimizing the microstructure of the hydrogel and forming a more ordered network, thus improving the material’s mechanical strength and stability.

### 3.6 Electrical conductivity

Conductive materials have vast prospects in bone tissue engineering, as they can significantly promote bone regeneration by regulating cell proliferation, differentiation, and mineralization. However, hydrogels typically exhibit poor mechanical strength and limited electrical conductivity (Li et al., 2022). Recent studies have found that MXenes not only sense cellular electrophysiology but also significantly enhance the electrical conductivity of hydrogels (Wang et al., 2024; Jervis et al., 2021). Hu et al. prepared an electroactive hydrogel based on regenerated silk fibroin and MXene, and found that the material showed good biocompatibility *in vivo* and animal experiments under electrical stimulation, and significantly promoted bone regeneration, mineralization, and angiogenesis. Through RNA sequencing, studies have shown that electrical stimulation can upregulate genes related to biomineralization, tissue development, and calcium signaling pathways, especially the CALM gene (calmodulin gene). This indicates that the osteogenic effect induced by electrical stimulation is closely related to the activation of Ca^2+^/CALM signaling in BMSCs (Hu et al., 2023). This study explored the effect of incorporating MXenes on the electrical conductivity of composite hydrogels. As shown in Figure 1M, the introduction of MXenes significantly modified the electrical conductivity of the hydrogels. Additionally, the conductivity increases further with higher concentrations of MXenes. The 0.5% MAG hydrogel group achieved a conductivity of 0.185 S/mm, categorizing it as a high-conductivity hydrogel. This enhanced conductivity is beneficial for promoting the proliferation and differentiation of osteoblasts, thereby facilitating bone regeneration.

### 3.7 *In vitro* cell biocompatibility studies

**FIGURE 2:**
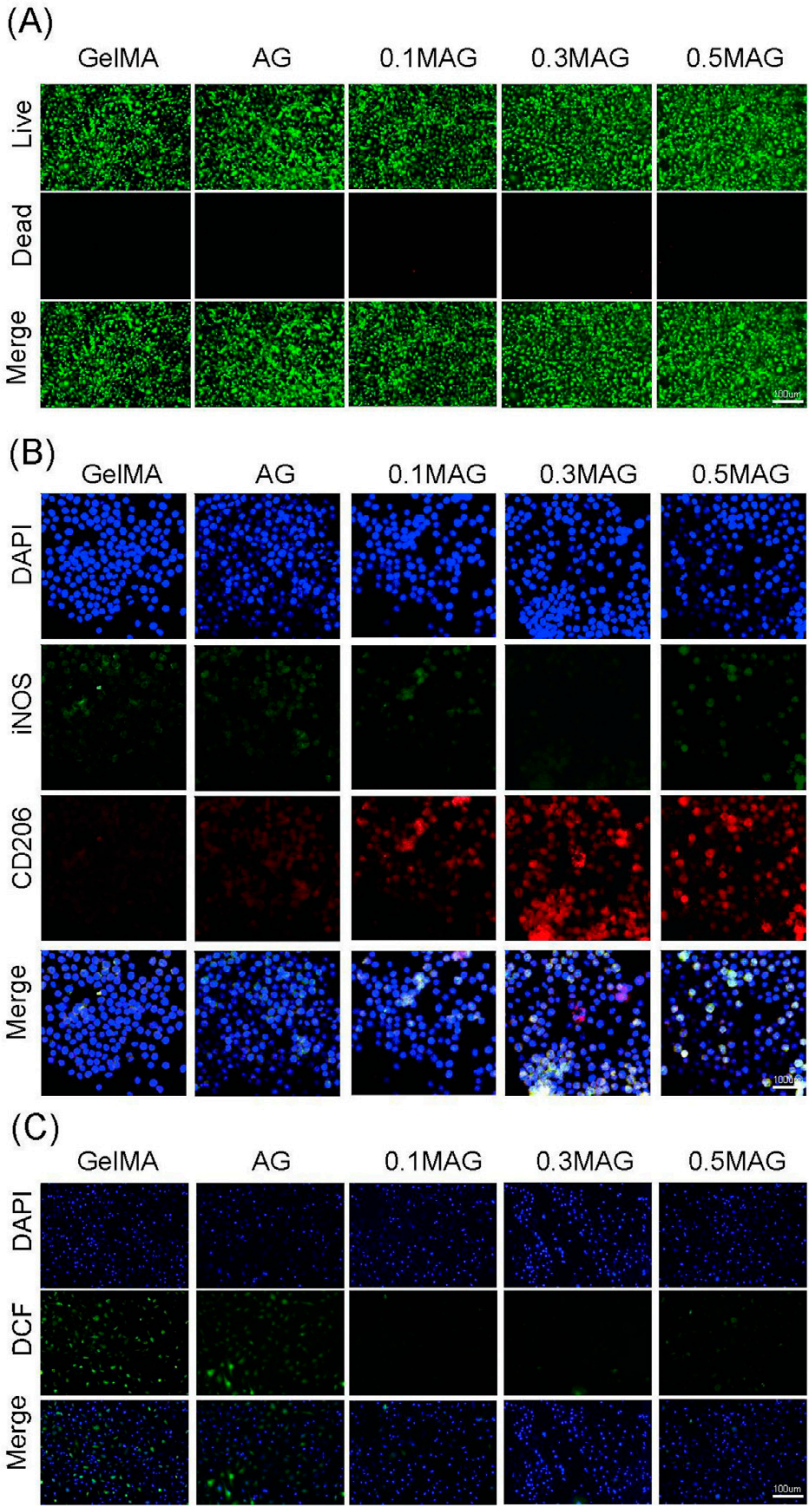
**(A)** Live (green)/dead (red) staining of MC3T3-E1 cells co-cultured with hydrogels for 4 days from each group; **(B)** Immunofluorescence staining of iNOS (green) and CD206 (red) in RAW264.7 cells co-cultured with hydrogels for 2 days from each group, the cell nuclei are labeled in blue; **(C)** ROS levels in MC3T3-E1 cells co-cultured with hydrogels for 2 days from each group, detected using the fluorescent probe DCFH-DA.

We further investigate the biocompatibility of each hydrogel group, as seen in Figure 2A, cells in all groups exhibited good growth, maintained high viability, and showed no significant cell death, indicating that all hydrogels had no obvious toxic effects.

To evaluate the anti-inflammatory properties, we performed fluorescence staining for iNOS and CD206 on the five sample groups. The results, shown in Figure 2B, indicated that the fluorescence intensity of iNOS in the 0.3% MAG group was remarkably lower, which suggested that an appropriate concentration of MXene helps to suppress the expression of the pro-inflammatory factor iNOS, thereby promoting tissue repair and regeneration. Furthermore, the fluorescence signal of CD206 in the 0.3% MAG group was observed to be significantly higher than in the other groups, implying the material facilitates the polarization of macrophages from the M1 to M2 type, which was beneficial for anti-inflammatory responses and indirect osteogenesis.

The antioxidant results, shown in Figure 2C, demonstrated that the ROS scavenging capacity of the MAG group was more preferable than that of the AG and GelMA group, while the 0.1% and 0.3% MAG groups displayed the best results. This indicated that the addition for low concentrations of MXene to the hydrogel aids in removing excess ROS, reducing oxidative stress, and maintaining extracellular matrix stability, thereby positively impacting the osteogenesis process.

**FIGURE 3:**
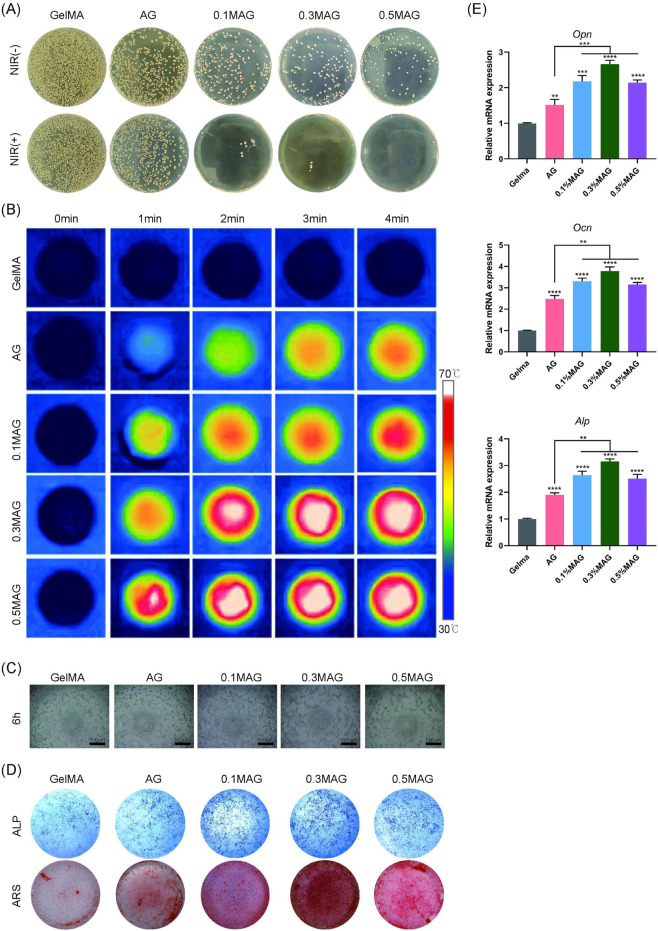
**(A)** Colony images showing the antibacterial effect of hydrogels from each group against *Staphylococcus aureus* without and with NIR illumination; **(B)** Infrared thermal images of hydrogels; **(C)** Angiogenesis of MC3T3-E1 cells co-cultured with hydrogels from each group at 6 h; **(D)** ALP staining at the seventh day and ARS staining at the 21st day of MC3T3-E1 cells co-cultured with hydrogels from each group; **(E)** PCR analysis of osteogenic-related genes OPN, OCN, and ALP expression in MC3T3-E1 cells co-cultured with hydrogels for 4 days from each group (****p < 0.0001; ***p < 0.001; **p < 0.01; *p < 0.05).

The healing of extraction sockets involves inflammation, cell proliferation, and tissue reconstruction, making it crucial to evaluate the antibacterial properties of bone tissue engineering scaffolds. We co-cultured the hydrogels with MC3T3-E1 cells and performed an antibacterial assay against *Staphylococcus aureus* using the plate coating method combined with NIR photothermal experiments. The results, shown in Figure 3A, indicated that the MAG hydrogels exhibited excellent antibacterial properties, which were further enhanced after NIR illumination. This antibacterial effect resulted from several factors: the mechanical disruption of bacterial membranes by nano-MXene, and the ability of MXene materials to efficiently convert light energy into heat energy under near-infrared light illumination (Avinashi et al., 2024). Through localized heating, MXene can effectively kill pathogenic microorganisms, thereby reducing the use of antibiotics and lowering the emergence of resistant strains (Seidi et al., 2023). The incorporation of low concentrations of Ag (Rai et al., 2009) also contributed to the antibacterial activity.

Moreover, this photothermal conversion can also promote bone regeneration. Local thermal stimulation enhances cellular metabolic activity, promotes the generation and mineralization of the extracellular matrix, and further accelerates bone tissue repair (Abdulghafor et al., 2024). We further explored the photothermal conversion capabilities of the composite hydrogels. As shown in Figure 3B, the addition of MXene endows the hydrogels with excellent photothermal conversion capabilities, allowing them to rapidly reach temperatures of 50°C–55°C under NIR illumination, thereby promoting the process of osteogenesis.

Angiogenesis is critical for wound healing and new bone formation. This study used angiogenesis assays to investigate the effects of each hydrogel group on promoting angiogenesis. As shown in Figure 3C, the 0.3% MAG hydrogel group had the highest amount of new blood vessels Additionally, we assessed the ability of the hydrogels to induce osteogenic differentiation and calcium deposition through ALP and ARS staining. The results shown in Figure 3D, suggest that, in compared with the GelMA group, the rest of four groups exhibited deeper staining, with the 0.3% MAG group showing the best results. RT-PCR was employed to measure the expression levels of osteogenesis-related genes ALP, OPN, and OCN. As shown in Figure 3E, the 0.3% MAG hydrogel significantly promoted osteogenesis by upregulating the expression of the osteogenic genes ALP, OPN, and OCN, thereby enhancing the proliferation, differentiation, and calcium deposition of osteoblasts.

**FIGURE 4:**
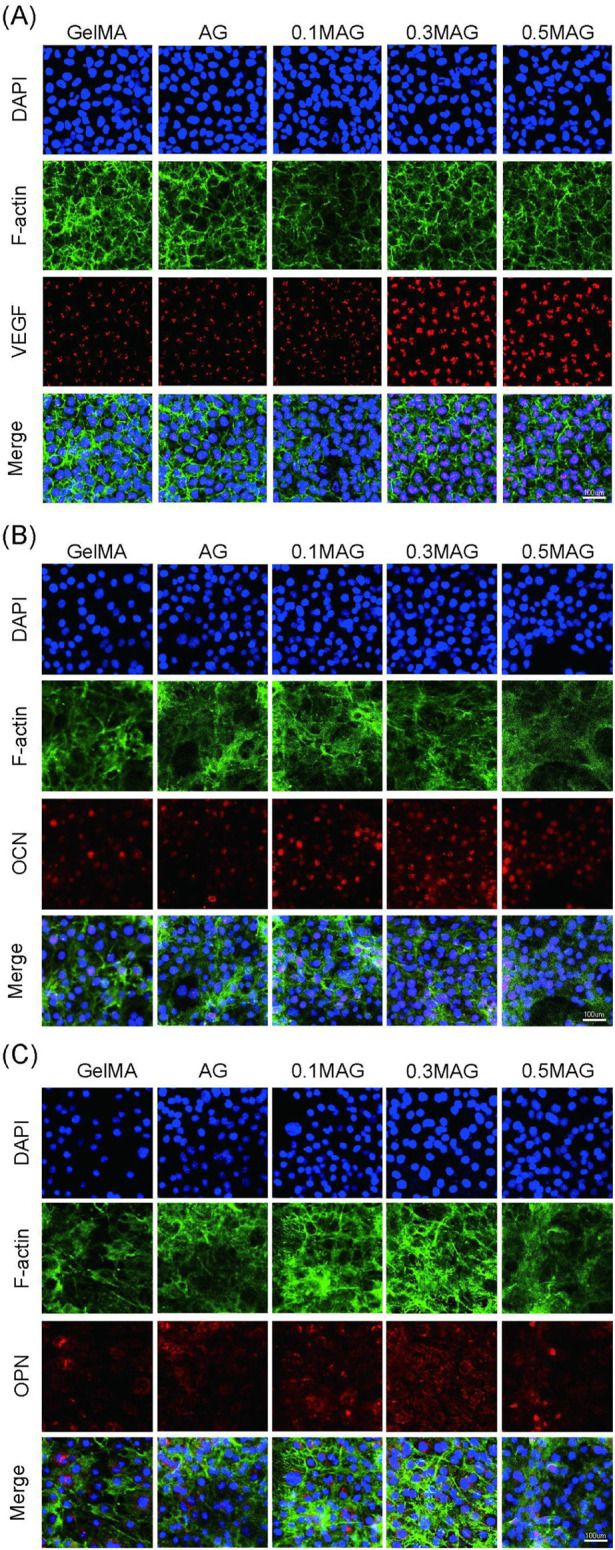
**(A)** Immunofluorescence staining of VEGF in MC3T3-E1 cells, **(B)** Immunofluorescence staining of OCN in MC3T3-E1 cells, and **(C)** Immunofluorescence staining of OPN in MC3T3-E1 cells co-cultured with hydrogels from each group at day 2, 4, and 4 respectively.

The results of immunofluorescence staining showed, as shown in Figure 4A, that the VEGF fluorescence signal was the strongest, indicating that an appropriate amount of MXene promotes the formation of new blood vessels and osteogenesis. The immunofluorescence staining for OPN and OCN further validated this result. As seen in Figures 4B, C, the 0.3% MAG group exhibited the strongest and most uniform fluorescence.

### 3.8 *In vivo* osteogenesis study

**FIGURE 5:**
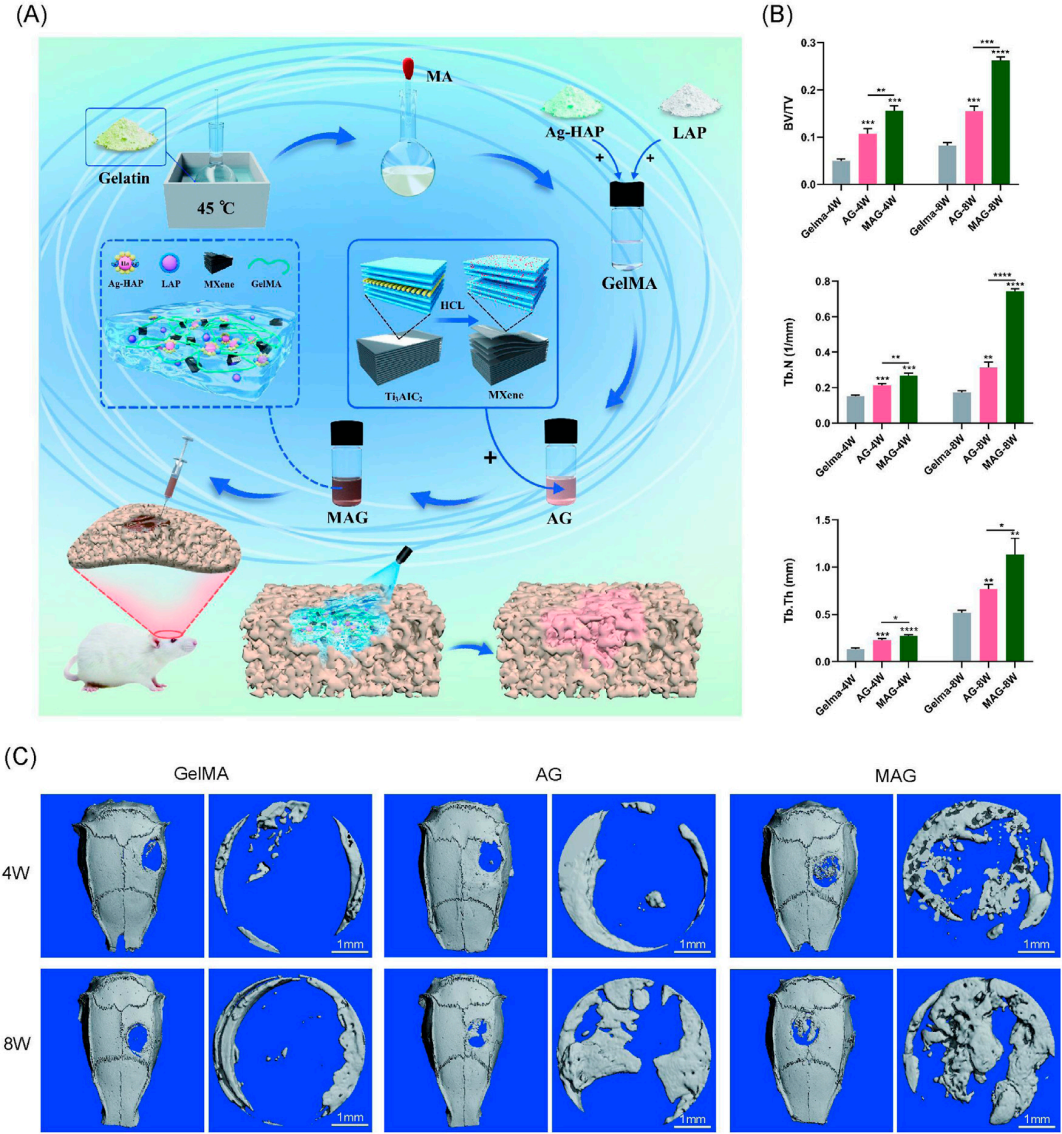
**(A)** Experimental design flowchart; **(B)** Quantitative analysis of bone volume fraction (BV/TV), trabecular number (Tb.N), and trabecular thickness (Tb.Th); **(C)** Micro-CT 3D reconstruction images of bone defect areas filled with hydrogels from each group at 4 and 8 weeks (****p < 0.0001; ***p < 0.001; **p < 0.01; *p < 0.05).

The overall design of the animal experiment is demonstrated in Figure 5A. Learning from the *in vitro* cell experiments, we selected the 0.3% MAG hydrogel group as the experimental group, with the AG group and GelMA hydrogel group as control groups for the animal experiments. At 4 and 8 weeks post-implantation into rat cranial bone defects, bone repair was observed via Micro-CT scanning. The results showed that the bone healing effects *in vivo* were highly consistent with the *in vitro* experiment results, with the MAG group demonstrating significant osteogenic advantages. This trend was also reflected in the trabecular number (Tb.N), bone volume fraction (BV/TV), and trabecular thickness (Tb.Th),as shown in Figure 5B.

At 4 weeks, the pure GelMA group exhibited the poorest bone repair, with only a few new bones forming at the edges of circular bone defect. The MAG group showed the best osteogenic effect, with the CT images revealing substantial bone bridging, indicating that the incorporation of MXene into the hydrogel promotes early new bone formation, consistent with the previous *in vitro* cell experiment results. At 8 weeks, the micro-CT results showed a similar trend, with the MAG group continuing to achieve the best bone repair, forming extensive new bone regeneration in the center of the defect, as shown in Figure 5C.

**FIGURE 6:**
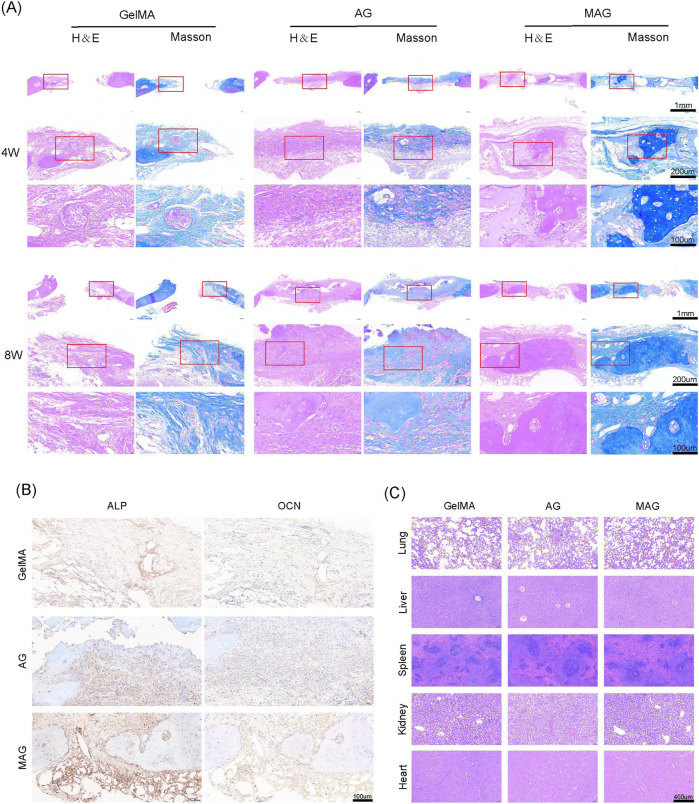
**(A)** HE and Masson staining of bone defect areas implanted with hydrogels from each group at 4 and 8 weeks; **(B)** ALP and OCN staining of bone defect areas implanted with hydrogels from each group at 8 weeks; **(C)** HE staining results of the heart, liver, spleen, lung, and kidney of rats after 8 weeks of hydrogel implantation in each group.

H&E staining, Masson staining, and immunohistochemical staining further confirmed these findings, as shown in Figures 6A, B. The inclusion of MXene promoted high expression of ALP and OCN, and the composite hydrogel exhibited extensive new bone formation around its periphery and in its gaps. Mature bone tissue formation and high collagen content were observed, demonstrating excellent bone integration and osteogenic potential, providing strong support for its future clinical applications. Meanwhile, the H&E staining results for various organs indicate that the MAG composite hydrogel has good biocompatibility *in vivo*, providing evidence for its safety in future clinical applications (Figure 6C).

### 3.9 Osteogenic gene mechanism study

In both of *in vitro* and *in vivo* studies, we demonstrated that the incorporation of MXene into hydrogels exhibited remarkable osteoinductive effects, showing significant advantages in bone regeneration and integration. In this study, we selected the 0.3% MAG group hydrogel as the experimental group, with AG hydrogel as the control group, and systematically analyzed the expression differences between these two composite hydrogels at the cellular and molecular levels. We delved into the osteogenic mechanism and signaling pathways of the MAG group, revealing its potential mechanisms in promoting bone regeneration.

**FIGURE 7:**
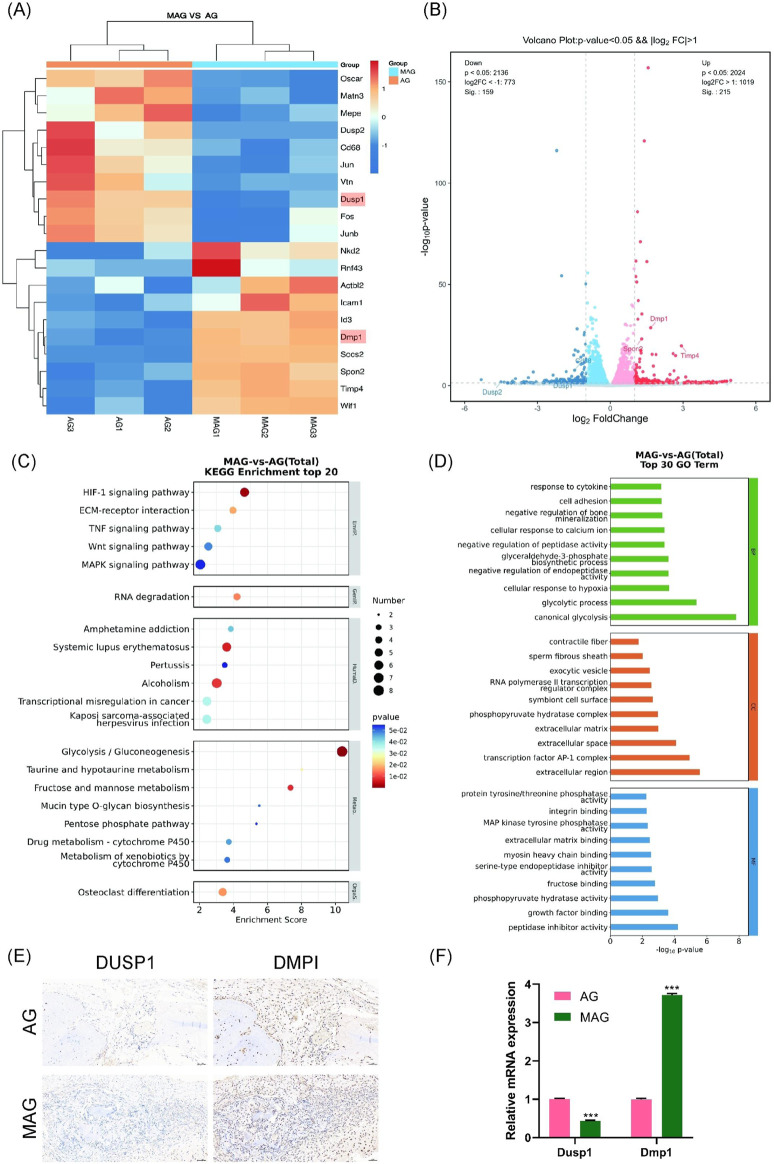
**(A)** Heatmap of differentially expressed gene expression levels; **(B)** Volcano plot of differentially expressed genes; **(C)** KEGG pathway enrichment analysis of differentially expressed genes; **(D)** GO enrichment analysis of differentially expressed genes between the groups; **(E)** DMP1 and DUSP1 staining of bone defect areas implanted with MAG and AG hydrogels at 8 weeks; **(F)** Verification of Dmp1 and Dusp1 Gene Expression Changes in MC3T3-E1 Cells Co-cultured with AG and MAG Hydrogels Using qPCR (*p < 0.001).

We set the significance level for differential gene selection at q < 0.05 and |log2(FC)| > 1. As illustrated in Figure 7A, the expression levels of these differentially expressed genes were analyzed using hierarchical clustering heatmaps. Compared to the AG group, the MAG group showed upregulated expression of non-collagenous matrix protein Dmp1, the MMP inhibition gene Timp4, and the cytoskeletal component ACTBL2. Moreover, the MAG group exhibited increased expression of genes related to cell movement (such as SPON2, Icam1, and Id3), which collectively enhanced osteogenesis.

Notably, the MAG group also significantly downregulated genes unfavorable to osteogenesis. For example, the expression of Dusp1, a negative regulator of the MAPK signaling pathway, was downregulated, which may enhance MAPK pathway activity. These findings were visually represented using volcano plots, highlighting gene expression differences between the two hydrogel materials (Figure 7B).

In the KEGG pathway analysis (Figure 7C), osteogenesis-related differential pathways were identified, including the Wnt and MAPK signaling pathways. Similarly, GO enrichment analysis (Figure 7D) indicated that MAG hydrogel could promote cell adhesion and calcium ion binding, enhance glycolysis to supply energy and metabolic intermediates, and regulate osteoblast proliferation, differentiation, and bone mineralization, thereby supporting osteoblast growth and function.

We selected Dmp1 and Dusp1 for experimental validation based on KEGG pathway analysis, GO enrichment analysis, and hierarchical clustering heatmaps, as these genes exhibited significant expression changes in the MAG group and played crucial roles in osteogenesis. Dmp1 activates the Wnt/β-catenin signaling pathway, regulating osteogenic genes such as Runx2 and OPN. Additionally, Dmp1 promotes osteoblast differentiation, contributing to bone mineralization and improving bone mechanical strength and stability (Kongkiatkamon et al., 2021). In contrast, Dusp1, a phosphatase that dephosphorylates and inactivates key molecules in the MAPK signaling pathway (Peng et al., 2017), such as ERK, JNK, and p38, was downregulated in the MAG group. This downregulation increases the activity of MAPKs, enhancing the transmission of the MAPK signaling pathway, and ultimately promoting osteoblast proliferation and differentiation.

As shown in Figure 7E, immunohistochemical staining in animal models was used to validate gene expression. The results demonstrated that the MAG hydrogel significantly upregulated Dmp1 expression while downregulating Dusp1 expression, thus facilitating osteoblast proliferation, differentiation, and mineralization. This trend was further confirmed by qPCR results (Figure 7F).

## 4 Conclusion

This study successfully prepared injectable MXene/Ag-HA composite hydrogel scaffolds. Physical characterization revealed uniform distribution of MXene in the composite hydrogel, demonstrating excellent mechanical properties, conductivity, and appropriate biodegradability of the scaffold. Through a series of *in vitro* and *in vivo* experiments, it was verified that the 0.3% concentration of MXene/Ag-HA hydrogel exhibits outstanding antibacterial properties, clears excessive ROS, induces macrophage polarization, promotes vascularization, and demonstrates excellent osteogenic capabilities. Genomic analysis revealed that MXene/Ag-HA upregulates Dmp1 and downregulates Dusp1 expression, activating the Wnt/β-catenin and MAPK signaling pathways, respectively, thereby regulating osteogenesis. Therefore, the studied MXene/Ag-HA composite hydrogel represents a promising bone repair material for use in bone regeneration and repair.

## Data Availability

The original contributions presented in the study are included in the article/supplementary material, further inquiries can be directed to the corresponding author.

## References

[B1] Abdulghafor M. A. Mahmood M. K. Tassery H. Tardivo D. Falguiere A. Lan R. (2024). Biomimetic coatings in implant dentistry: a quick update. J. Funct. Biomater. 15, 15. 10.3390/jfb15010015 PMC10816551 38248682

[B2] Avinashi S. K. Mishra R. K. Singh R. Fatima Z. Gautam C. R. (2024). Fabrication methods, structural, surface morphology and biomedical applications of MXene: a review. ACS Appl. Mater. Interface 16, 47003–47049. 10.1021/acsami.4c07894 39189322

[B3] Daniel Arcos D. Vallet-Regí M. (2020). Substituted hydroxyapatite coatings of bone implants. J. Mater. Chem. B 8, 1781–1800. 10.1039/C9TB02710F 32065184 PMC7116284

[B4] Faour O. Dimitriou R. Cousins C. A. Giannoudis P. V. (2011). The use of bone graft substitutes in large cancellous voids: any specific needs? Injury 42, 87–S90. 10.1016/j.injury.2011.06.020 21723553

[B5] Gazzi A. Fusco L. Khan A. Bedognetti D. Zavan B. Vitale F. (2019). Photodynamic therapy based on graphene and MXene in cancer theranostics. Front. Bioeng. Biotech. 7, 295. 10.3389/fbioe.2019.00295 PMC6823231 31709252

[B6] Hu Z. Lu J. Zhang T. Liang H. Yuan H. Su D. (2023). Piezoresistive MXene/silk fibroin nanocomposite hydrogel for accelerating bone regeneration by Re-establishing electrical microenvironment. Bioact. Mater. 22, 1–17. 10.1016/j.bioactmat.2022.08.025 36203961 PMC9513113

[B7] Iocca O. Farcomeni A. Lopez S. P. Talib H. S. (2017). Alveolar Ridge preservation after tooth extraction: a bayesian network meta-analysis of grafting materials efficacy on prevention of bone height and width reduction. J. Clin. Periodontol. 44, 104–114. 10.1111/jcpe.12633 27712001

[B8] Jervis P. J. Amorim C. Pereira T. Martins J. A. Ferreira P. M. T. (2021). Dehydropeptide supramolecular hydrogels and nanostructures as potential peptidomimetic biomedical materials. Int. J. Mol. Sci. 22, 2528. 10.3390/ijms22052528 33802425 PMC7959283

[B9] Kongkiatkamon S. Ramachandran A. Knoernschild K. L. Campbell S. D. Sukotjo C. George A. (2021). Dentin matrix protein 1 on titanium surface facilitates osteogenic differentiation of stem cells. Molecules 26, 6756. 10.3390/molecules26226756 34833848 PMC8621853

[B10] Kurian A. G. Singh R. K. Patel K. D. Lee J.-H. Kim H.-W. (2022). Multifunctional GelMA platforms with nanomaterials for advanced tissue therapeutics. Bioact. Mater. 8, 267–295. 10.1016/j.bioactmat.2021.06.027 34541401 PMC8424393

[B11] Li J. Gao L. Xu R. Ma S. Ma Z. Liu Y. (2022). Fibers reinforced composite hydrogels with improved lubrication and load-bearing capacity. Friction 10, 54–67. 10.1007/s40544-020-0389-9

[B12] Li T. Zeng X. Zou S. Xu Y. Duan P. (2023). Recent advances in horizontal alveolar bone regeneration. Biomed. Mater. 18, 052004. 10.1088/1748-605X/acd672 37196651

[B13] Li Y. Yang H. Lee D. (2021). Advances in biodegradable and injectable hydrogels for biomedical applications. J. Control Release 330, 151–160. 10.1016/j.jconrel.2020.12.008 33309972

[B14] Lin H. Chen Y. Shi J. (2018). Insights into 2D MXenes for versatile biomedical applications: current advances and challenges ahead. Adv. Sci. 5, 1800518. 10.1002/advs.201800518 PMC6193163 30356929

[B15] Mirzaee M. Vaezi M. Palizdar Y. (2016). Synthesis and characterization of silver doped hydroxyapatite nanocomposite coatings and evaluation of their antibacterial and corrosion resistance properties in simulated body fluid. Mater. Sci. Eng. 69, 675–684. 10.1016/j.msec.2016.07.057 27612761

[B16] Parajuli D. Murali N. Kc D. Karki B. Samatha K. Kim A. (2022). Advancements in MXene-polymer nanocomposites in energy storage and biomedical applications. Polymers 14, 3433. 10.3390/polym14163433 36015690 PMC9415062

[B17] Peng H. Yun Z. Wang W. Ma B. (2017). Dual specificity phosphatase 1 has a protective role in osteoarthritis fibroblast-like synoviocytes via inhibition of the MAPK signaling pathway. Mol. Med. Rep. 16, 8441–8447. 10.3892/mmr.2017.7617 28983624

[B18] Rai M. Yadav A. Gade A. J. Ba. (2009). Silver nanoparticles as a new generation of antimicrobials. Biotechnol. Adv. 27, 76–83. 10.1016/j.biotechadv.2008.09.002 18854209

[B19] Ramezanzade S. Aeinehvand M. Ziaei H. Khurshid Z. Keyhan S. O. Fallahi H. R. (2023). Reconstruction of critical sized maxillofacial defects using composite allogeneic tissue engineering: systematic review of current literature. Biomimetics 8, 142. 10.3390/biomimetics8020142 37092394 PMC10123735

[B20] Ren S. Lin Y. Liu W. Yang L. Zhao M. (2023). MSC-exos: important active factor of bone regeneration. Front. Bioeng. Biotechnol. 11, 1136453. 10.3389/fbioe.2023.1136453 36814713 PMC9939647

[B21] Seidi F. Shamsabadi A. A. Firouzjaei M. D. Elliott M. Saeb M. R. Huang Y. (2023). MXenes antibacterial properties and applications: a review and perspective. Small 19, 2206716. 10.1002/smll.202206716 36604987

[B22] Shi H. Zhou Z. Li W. Fan Y. Li Z. Wei J. (2021). Hydroxyapatite based materials for bone tissue engineering: a brief and comprehensive introduction. Crystals 11, 149. 10.3390/cryst11020149

[B23] Sinha A. Zhao H. Huang Y. Lu X. Chen J. Jain R. (2018). MXene: an emerging material for sensing and biosensing. Trac-Trend Anal. Chem. 105, 424. 10.1016/j.trac.2018.05.021

[B24] Toosi S. Javid-Naderi M. J. Tamayol A. Ebrahimzadeh M. H. Yaghoubian S. Mousavi Shaegh S. A. (2024). Additively manufactured porous scaffolds by design for treatment of bone defects. Front. Bioeng. Biotechnol. 11, 1252636. 10.3389/fbioe.2023.1252636 38312510 PMC10834686

[B25] Wang B. Feng C. Liu Y. Mi F. Dong J. (2022). Recent advances in biofunctional guided bone regeneration materials for repairing defective alveolar and maxillofacial bone: a review. Jpn. Dent. Sci. Rev. 58, 233–248. 10.1016/j.jdsr.2022.07.002 36065207 PMC9440077

[B26] Wang H. Hsu Y.-C. Wang C. Xiao X. Yuan Z. Zhu Y. (2024). Conductive and enhanced mechanical strength of Mo_2_Ti_2_C_3_ MXene-based hydrogel promotes neurogenesis and bone regeneration in bone defect repair. ACS Appl. Mater. Interfaces 16, 17208–17218. 10.1021/acsami.3c19410 38530974

[B27] Wang H. Mu N. He Y. Zhang X. Lei J. Yang C. (2023). Ultrasound-controlled MXene-based Schottky heterojunction improves anti-infection and osteogenesis properties. Theranostics 13, 1669–1683. 10.7150/thno.81511 37056559 PMC10086208

[B28] Ye S. Zhang H. Lai H. Xu J. Yu L. Ye Z. (2024). MXene: a wonderful nanomaterial in antibacterial. Front. Bioeng. Biotechnol. 12, 1338539. 10.3389/fbioe.2024.1338539 38361792 PMC10867285

[B29] Yin J. Pan S. Guo X. Gao Y. Zhu D. Yang Q. (2021). Nb_2_C MXene-functionalized scaffolds enables osteosarcoma phototherapy and angiogenesis/osteogenesis of bone defects. Nano-Micro Lett. 13, 30. 10.1007/s40820-020-00547-6 PMC8187678 34138204

[B30] Yu F. Lian R. Liu L. Liu T. Bi C. Hong K. (2022). Biomimetic hydroxyapatite nanorods promote bone regeneration via accelerating osteogenesis of BMSCs through T cell-derived IL-22. ACS Nano 16, 755–770. 10.1021/acsnano.1c08281 35005890

[B31] Yue K. Santiago G. T. Alvarez M. M. Tamayol A. Annabi N. Khademhosseini A. (2015). Synthesis, properties, and biomedical applications of gelatin methacryloyl (GelMA) hydrogels. Biomater 73, 254. 10.1016/j.biomaterials.2015.08.045 PMC4610009 26414409

[B32] Zhang Y. Wu D. Zhao X. Pakvasa M. Tucker A. B. Luo H. (2020). Stem cell-friendly scaffold biomaterials: applications for bone tissue engineering and regenerative medicine. Front. Bioeng. Biotechnol. 8, 598607. 10.3389/fbioe.2020.598607 33381499 PMC7767872

[B33] Zhao R. Xie P. Zhang K. Tang Z. Chen X. Zhu X. (2017). Selective effect of hydroxyapatite nanoparticles on osteoporotic and healthy bone formation correlates with intracellular calcium homeostasis regulation. Acta Biomater. 59, 338–350. 10.1016/j.actbio.2017.07.009 28698163

